# Feasibility study of an optimised person-centred intervention to improve mental health and reduce antipsychotics amongst people with dementia in care homes: study protocol for a randomised controlled trial

**DOI:** 10.1186/1745-6215-14-13

**Published:** 2013-01-10

**Authors:** Rhiannon Whitaker, Clive Ballard, Jane Stafford, Martin Orrell, Esme Moniz-Cook, Robert T Woods, Joanna Murray, Martin Knapp, Barbara Woodward Carlton, Jane Fossey

**Affiliations:** 1 North Wales Organisation for Randomised Trials in Health, Bangor University, Holyhead Road, Bangor LL57 2PZ Gwynedd, United Kingdom; 2 Wolfson Centre for Age Related Diseases, King's College London, Guy's Campus, London SE1 1UL, United Kingdom; 3 Psychological Services, Oxford Health NHS Foundation Trust, Fulbrook Centre, Oxford OX3 7JU, United Kingdom; 4 University College London, 67-73 Riding House Street, London, W1W 7EJ, United Kingdom; 5 Institute of Rehabilitation: Dementia Applied Research Centre, University of Hull, Health House, Grange Park Lane, East Yorkshire HU10 6DT, United Kingdom; 6 Dementia Services Development Centre, Wales, Institute of Medical and Social Care Research, Bangor University, Holyhead Road, Bangor LL57 2PX Gwynedd, United Kingdom; 7 Section of Mental Health and Ageing, Health Service and Population Research Department, The Institute of Psychiatry at King's College London, PO26, The David Goldberg Centre, De Crespigny Park, London, SE5 8AF, United Kingdom; 8 London School of Economics and Political Science, Houghton Street, London WC2A 2AE, United Kingdom; 9 Alzheimer's Society, Devon House, 58 St Katharine's Way, London E1W 1LB, United Kingdom

**Keywords:** Dementia, Care homes, Quality of life, Antipsychotic medication, Behavioural symptoms, Cost-effectiveness, Implementation, Person-centred care, Exercise, Social interaction

## Abstract

**Background:**

People living in care homes often have complex mental and physical health problems, disabilities and social needs which are compounded by the use of psychiatric and other drugs. In the UK dementia care is a national priority with a vast impact on services. WHELD combines the most effective elements of existing approaches to develop a comprehensive but practical intervention. This will be achieved by training care staff to provide care that is focused on an understanding of the individual and their needs; and by using additional components such as exercise, activities and social interaction to improve mental health and quality of life (QoL) and reduce the use of sedative drugs.

**Design:**

Work Package 3 (WP3) is the pilot randomised trial and qualitative evaluation to help develop a future definitive randomised controlled clinical trial. The study design is a cluster randomised 2x2x2 factorial design with two replications in 16 care homes. Each care home is randomized to receive one of the eight possible permutations of the four key interventions, with each possible combination delivered in two of the 16 homes. Each cluster includes a minimum of 12 participants (depending upon size of the care home, the number of people with dementia and the number consenting).

**Discussion:**

The overarching goal of the programme is to provide an effective, simple and practical intervention which improves the mental health of, and reduces sedative drug use in, people with dementia in care homes and which can be implemented nationally in all UK care homes as an NHS intervention.

**Trial Registration:**

Current controlled trials ISRCTN40313497

## Background

Twenty five million people worldwide have dementia, including 700,000 in the UK [1], of whom an estimated 250,000 live in care homes [1,2]. Older people with dementia in care homes have complex needs; for example, cognitive and functional impairment often coexists with additional mental health problems such as aggression, agitation, depression and psychosis [3]. These difficulties are further compounded by the widespread prescription of antipsychotic drugs [2,4]. The cost of prescription of antipsychotic medication is estimated to be £84 million a year for 140,000 people in the UK, who are unlikely to benefit and may be harmed by them [5].

Dementia has a vast impact on Health and Social Care Services. The direct cost of Alzheimer’s disease is £17 billion per year [1], greater than for stroke, heart disease and cancer combined. The high level of unmet need and the management of key health and mental health issues are matters for serious concern both nationally [6], and internationally [7]. In the UK this led to the National Dementia Strategy (NDS) [8], which was developed as a partnership between the Department of Health (DH) and key stakeholders such as the Alzheimer’s Society. It is a unique vision for people with dementia and provides a five-year plan to ‘develop services for people with dementia and their carers that are fit for the 21st century and that meet the needs of everyone’. Key goals include objective number 11 ‘Improving the quality of care for people with dementia in care homes’, achieved through a variety of measures, including visits from specialist mental health teams, and objective 13, the development of an ‘informed and effective workforce for people with dementia’, whereby health and social care staff will receive the right training and have the right skills to deliver the best care.

The National Service Framework (NSF) for older people [9], the National Institute for Clinical Excellence (NICE) dementia guidelines [10] and a market analysis [11,12] also highlight the importance of training for care staff and the need to improve access to effective non-pharmacological therapies. The DH has also conducted a review of antipsychotic prescribing [13,14] for people with dementia, which recommends a substantial reduction in unnecessary prescribing. This adds further weight to the recommendations of the NDS, the NSF and the NICE dementia guidelines to improve the treatment and care for people with dementia in care homes. Care home regulators in the US have launched initiatives to tackle the same key issues [15]. The NDS vision of enabling people with dementia in care homes to live well with dementia needs to be underpinned by effective, evidence-based interventions that are standardized, consistent, practical and can be delivered as part of the National Health Service (NHS).

Improving the care of mental health problems, reducing antipsychotic use and improving quality of life for people with dementia in care homes are all key NHS priorities. There is strong evidence that staff training, promoting person-centred care (PCC) and utilising non-pharmacological interventions improve some key health outcomes and can reduce antipsychotic drug use [10,16]. However, the breadth of benefit conferred by most of these interventions is modest; none have directly improved the quality of life (QoL) for care home residents with dementia, and importantly, none have achieved widespread implementation in a health or care setting as part of routine NHS practice.

Further research is therefore urgently needed to address these key issues. There is a need for an optimised therapy combining the most effective elements of currently available evidence-based interventions that are conceptually integrated, cost-effective and practical to implement in a range of settings. To achieve these objectives there is a need to evaluate the key components of effective interventions to enable the development of an optimised intervention.

Overcoming the barriers to implementation, which is rare in dementia care research and has never been achieved for any therapy in a care home setting, is also a major challenge. Research is needed to tailor an optimised therapy to the needs of mental health practitioners, care home staff and people with dementia in care homes. In addition, research should identify and overcome the potential obstacles to implementation, and refine the intervention model through in-depth field testing. This programme aims to provide an effective, simple and practical intervention that improves mental health and reduces sedative drug use for people with dementia in care homes, and which is replicable and scalable to enable it to be widely used in the UK and internationally. The current paper describes the pilot study and qualitative evaluation, setting the foundation for the full-scale multicentre randomised controlled clinical trial to evaluate an optimised intervention ‘welding together’ the most effective elements of the best currently available intervention programmes and a standardised manual and training programme (WHELD).

### Study objectives

The pilot study follows a factorial randomised controlled clinical trial design. Evaluations will be undertaken to understand the breadth of additional benefits conferred by three interventions, namely, antipsychotic review (discontinuation and safety); social interaction and pleasant activities; and exercise, compared with PCC alone.

### Hypotheses

Each intervention will significantly improve several key outcomes but none when used alone will improve all outcomes. This pilot randomised trial is not powered to provide a definitive answer but to guide the analysis and to generate firm hypotheses for testing in a definitive large-scale cluster randomised trial.

Specifically the hypotheses are that compared to person-centred care alone are:

1. PCC plus antipsychotic review will result in the reduction of antipsychotic prescribing.

2. PCC and social interaction with pleasant activities will result in reductions in agitation/aggression, especially in individuals already experiencing these symptoms at the baseline evaluation.

3. PCC and exercise will improve mood and reduce falls.

### Secondary objectives and qualitative evaluation

To inform subsequent work, the key secondary objectives are to determine the specific impact of each therapy on a range of outcomes including, mental health, psychotropic drug use, physical health and QoL, as well as the impact on potentially important mediating factors such as activities, social interaction, staff attitudes and the quality of the interaction between care staff and people with dementia.

The purpose of the qualitative research is to develop understanding of the process of implementation within the care environment. Staff beliefs, attitudes and behaviour in their work with people with dementia are key components. Recognition and acknowledgement of staff perspectives is also essential to negotiating the implementation of the interventions.

## Methods/Design

### Overall design

**Figure 1:**
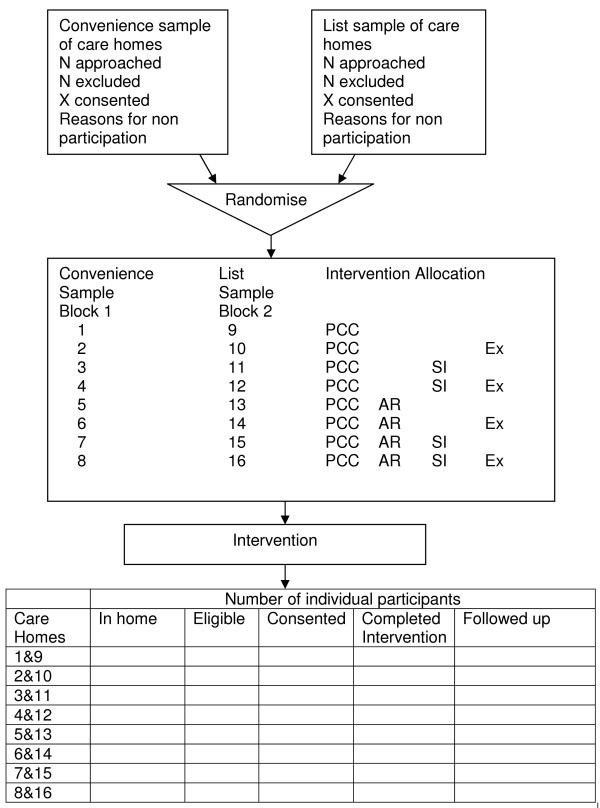
Design and consort diagram.

**Figure 2:**
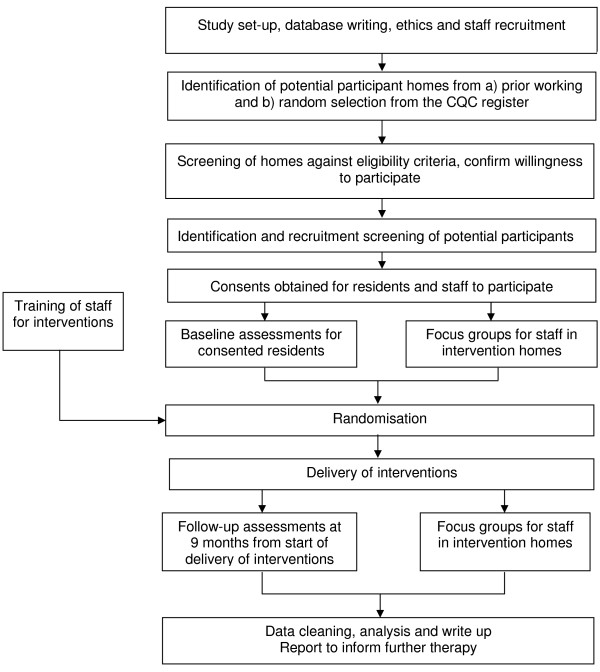
Flow chart for work plan 3.

The study design is a cluster-randomised, 2 × 2 × 2 factorial design (with two replications), pilot study in 16 care homes, as shown in Figure 1. Each cluster includes a minimum of 12 participants (depending upon the size of the care home, the number of people with dementia and the number consenting). Each cluster receives a randomly allocated intervention for a minimum of nine months. Evaluations will be undertaken to understand the breadth of benefits conferred by three key interventions to be assessed when used in addition to the person-centred care training package, where efficacy has already been established. The flow chart for the research is shown in Figure 2.

The WHELD PCC intervention primarily uses the tools developed in evidence-based approaches for improving care in care homes in the operationalized *Focussed Intervention, Training and Support* (FITS) manual [17], which has demonstrated efficacy in a robust randomised controlled trial (RCT) [18]. Additional supplementary materials have been drawn from the best available training manuals from a robust review of available materials conducted as part of the WHELD study, and augmented by elements of leadership training on the basis of input from an expert therapy development group. The intervention has 5 focii:

1. Embedding an understanding of dementia and PCC.

2. Assessing how home practices deliver PCC.

3. Understanding the relationship between an individuals’ experience and individuals’ behaviour and well being.

4. Recognising the impact of staff-resident interactions on the care experience.

5. Implementing PCC planning based on these principles.

This training package will be delivered to all staff in the participating care homes.

Antipsychotic review will involve specific review of antipsychotic drugs by participants’ own General Practitioners (GPs) or psychiatry specialists, based upon the principles outlined in the NICE dementia guidelines, and facilitated by an antipsychotic care pathway developed by the Alzheimer’s Society in partnership with the DH [13]. GPs will be offered an initial seminar outlining the best practice guidelines, and prompted when 12-week antipsychotic reviews are due according to NICE/Social Care Institute for Excellence (SCIE) guidelines. Care home staff will be offered a seminar on safe prescribing, monitoring and review of antipsychotic drugs. In addition, for all participants continuing to receive antipsychotic drugs after the initial review, or where antipsychotic drugs are started or re-started, a detailed medical antipsychotic care plan will be advised, using the principles outlined in the antipsychotic care pathway. This will include planned dates for further antipsychotic review.

For social interaction with pleasant activities, an intervention manual will be developed based upon three evidence-based approaches and specific communication skills training to enhance staff-resident interactions: (1) the positive events schedule, shown to be effective in the treatment of agitation and depression in people with dementia in non-care home settings [19,20]; (2) the social interaction intervention developed by Cohen-Mansfield and colleagues [21], which is useful for reducing agitation in people with dementia in care homes; and (3) the Needs, Environment, Stimulation, and Technique (NEST) programme, developed by Buettner and colleagues [22], which has been shown to be effective in improving cognitive function and reducing agitation and depression. Minor adaptations will be undertaken, in collaboration with the authors who developed the manuals, to ensure that they are suitable and practical for administration in UK care homes.

For exercise, the main focus will be to promote exercise through encouraging enjoyable positive physical activities. Teri and colleagues have developed an effective approach, based on their positive event schedule but focused on exercise-based activities [20]. The NEST manual [22] and the Range of Motion (ROM) dance programme [23], which an RCT has shown to be effective for older people with arthritis in care settings [24], will be used as specific resources to offer people enjoyable individual and group exercise activities to augment activities identified as hobbies or enjoyable activities by individual participants.

The factorial design is shown below. For example, care homes 1 and 9 will receive PCC only, while care home 4 will receive social interaction and exercise in addition to PCC and care home 13 will only receive antipsychotic review in addition to PCC. Staff will receive training in the interventions allocated to the care home, with all care homes receiving training in the PCC intervention.

Each intervention is delivered by two trained therapists, who have received an intensive 10-day training package and who will coordinate the delivery of the intervention into eight care homes. In each care home two lead members of the care staff (WHELD champions) will be trained to implement the intervention.

### Number of participants and power of the study

Sixteen suitable care homes to be identified, recruited and randomised, with the intervention delivered to all residents, with a minimum recruitment target of 12 individuals with dementia per care home. All residents within the care homes, who meet the study’s eligibility criteria will be invited to participate, which will help to account for the potential loss of participants during the course of the study. Baseline and follow up data will be collected on all consenting residents who meet the inclusion criteria at each participating care home. This is an exploratory pilot study whose main purpose is to collect data to enable the design and sample size calculation for the definitive RCT. As such, the size of effect for the outcome measures, their SDs and intra-class correlations (ICCs) are unknown.

### Randomisation

A restricted randomisation method will allocate the eight interventions to the eight care homes in the two samples. The randomisation will be performed as a constrained complete list randomisation, meaning that all care homes will have been recruited before the randomisation is performed. The constraint ensures an approximately equal distribution of the number of interventions to each geographic location. The randomisation system used has been coded and validated in R (statistical package). The R Foundation for Statistical Computing c/o Institute for Statistics and Mathematics, Vienna, Austria.

### Qualitative evaluation

The qualitative research will increase the understanding of the process of implementation within the environment in which the interventions take place. Focus groups (FGs) have been chosen to investigate the perspectives of care staff within the setting in which the interventions will be tested. The aim of FGs is to encourage the type of interaction that would occur in everyday life but with greater focus, and to allow the researchers access to ideologies, practices and desires among specific groups of people. Engaging groups of care staff in discussion of their current work and new therapeutic approaches is also likely to increase their collaboration in the research programme. Staff beliefs, attitudes and behaviour in their work with people with dementia are key components of this context. Recognition and acknowledgement of staff perspectives is also essential to negotiating the implementation of the interventions.

The first phase of the qualitative evaluation will be undertaken in the care homes (residential and nursing homes) participating in the intervention study, prior to the commencement of the intervention. The pre-intervention groups at the sixteen participating care homes will consist of eight to twelve members of the care staff, including the two lead members of the care staff who are to be trained to assist with the implementation of the intervention. Evidence from the systematic review will provide the initial themes to be explored in the FGs. Potential issues may, for example, include staff time to implement interventions and management support. The topic guide will be revised iteratively to include new themes emerging from the FGs. Participants will be encouraged to discuss their work with residents with dementia, their perceptions of residents’ quality of life, unmet needs and ways to improve these, the potential benefits of the intervention and their own role in implementation. The aims and content of the specific interventions will be considered.

The FGs will be repeated at the end of the pilot study to explore participants’ experiences of the interventions and any changes in their views. The researcher will provide a summary of the FG’s first discussion and seek feedback on interpretation and any changes in views post intervention. They will be asked to discuss positive and negative outcomes, obstacles and facilitators in delivering the intervention. Their views on refining the interventions will be sought. All group discussions will be audio-recorded and transcribed verbatim.

### Intervention fidelity

To monitor intervention fidelity, each therapist will be required to videotape at least two interventions and/or planning sessions. In addition, the type and frequency of interventions will be determined through audit of an intervention log completed by the therapist; audit of detailed intervention plans describing each intervention undertaken; audit of care plans; and a supervision log. In particular, given that there is a degree of overlap between the social interaction and pleasant activities intervention and the exercise intervention, it will be especially important to ensure that the exercise intervention specifically promotes exercise-based activities and to clearly profile the similarities and differences in these two intervention packages.

### Economic evaluation

The economic evaluation in this pilot study will focus on the amount of staff time needed and the overall cost of each intervention, as well as the patterns of service use for individual residents and the associated costs. It will do this by collecting information on the current living arrangements (including usual place of residence and other places of residence); followed by questions about any use the participant may have made of a range of hospital, community-based and day services (both health and social care) over a defined retrospective period. These data on service contacts, staff time and costs will be examined alongside data on outcomes to help preparation for the main study later in the WHELD programme.

### Inclusion, exclusion and withdrawal criteria

#### Home selection: inclusion, exclusion and withdrawal criteria

Eight care homes will represent a convenience sample (block 1) of local care homes, already known to the research team, which meet the inclusion and exclusion criteria and have previously expressed a willingness to participate in research. The other eight care homes will be identified from all care homes in the research area rated as ‘adequate’ or better on the Care Quality Commission (CQC) register (block 2). All care homes listed in the CQC register that meet the study’s inclusion and exclusion criteria will be entered into a study database. Next, this list of eligible care homes will be randomised and the homes approached in the order of appearance on the randomised list. If a care home declines to participate the next care home on the list will be approached.

Care homes scored adequate or better on the CQC register will be eligible for inclusion. Care homes in which 60% or fewer of the residents have dementia, and care homes receiving special support from a local authority will be excluded.

### Participant selection: inclusion, exclusion and withdrawal criteria

All residents who potentially eligible for evaluation will be identified by the care home staff. The inclusion criteria will be individuals residing in participating care homes and meeting the diagnostic criteria for dementia, a score of 1 or greater on the Clinical Dementia Rating Scale (CDR) [25], and a score of 4 or greater on the functional assessment staging (FAST) [26]. Any resident for whom consent is not obtained will be excluded. In terms of withdrawal criteria, individual participants would be able to withdraw from the study evaluation at any time.

### Staff selection: inclusion, exclusion and withdrawal criteria

All staff working in participating care homes would be potentially eligible to participate in the FGs as part of the qualitative evaluation. Consent for their participation will be sought separately. They will be excluded if consent is not obtained and are able to withdraw from the study at any time.

### Assessment and follow up

Assessments will be made pre-baseline to assess the suitability of the care homes for inclusion, as shown in Table 1. This process will include assessments to identify the total number of participants likely to be eligible for screening. Participants will be screened and consent obtained prior to baseline evaluation and to randomization of the care homes. Follow up assessments will be made nine months after commencement of the intervention.

**Table 1:** Outcome and study eligibility measures

	**Domain**	**Purpose**	**How long to complete**	**Who completes**	**Pre-BL**	**BL**	**Ongoing**	**Post-int**
**Measures of home**								
Number and approximate proportion of residents with dementia	Demographic	Eligibility	1 hour - records search and informant interview	Researcher	Y			Y
Rating on CQC criteria	Demographic	Eligibility	15 mins - records search	Researcher	Y			Y
**Measures of individual**								
Demographics	Demographic	Demographics	10 mins - records search and informant interview	Researcher		Y		Y
Functional assessment staging (FAST) [26]	Diagnostic criteria	Eligibility	15 mins trial assessments and informant interview	Researcher	Y			Y
CDR [25]	Diagnostic criteria	Eligibility	15 mins - trial assessments and informant interview	Researcher	Y			
Antipsychotic use (number and proportion of people and dose)	Medication use	Quantitative	5 mins per resident - records search and drug chart	Researcher		Y		Y
		Primary outcome						
Use of other psychotropic drugs (No. and proportion of people and dose)	Medication use	Quantitative	5 mins per resident - records search and drug chart	Researcher		Y		Y
		Secondary outcome						
Cohen-Mansfield agitation inventory (CMAI) [27]	Agitation	Quantitative	10 mins	Researcher with staff		Y		Y
		Primary outcome						
Neuropsychiatric inventory - nursing home version (NPI-NH) [28]	Other behavioural and neuro-psychiatric symptoms including apathy and psychosis	Quantitative	20 mins	Researcher with staff		Y		Y
		Secondary outcome						
Cornell depression scale [29]	Mood	Quantitative	10-15 mins	Researcher with staff and residents		Y		Y
		Secondary outcome						
Rating anxiety in dementia (RAID) [30]	Mood	Quantitative	10-15 mins each	Researcher with staff and residents		Y		Y
		Secondary outcome						
Camberwell assessment of need in the elderly (CANE) [31]	Unmet needs	Quantitative	30 mins each	Researcher with staff and residents		Y		Y
		Secondary outcome						
Assessment of QoL for people with dementia (DEMQOL) [32]	Quality of life (QoL)	Quantitative	20 mins each	Researcher with staff and separately with residents		Y		Y
		Secondary Outcome						
QoL in Alzheimer’s disease (QoL-AD) [33]	Quality of life	Quantitative	5-10 mins	Researcher with staff and separately with residents		Y		Y
		Secondary Outcome						
Quality of interaction schedule (QUIS, observational tool) [34]	Quality of interactions between staff and residents	Quantitative	2 hours observation per home	Researcher		Y		Y
		Secondary Outcome						
Falls record	Falls	Quantitative	5 mins	Care staff			Y	
		Secondary outcome						
Case examples	Skills and attitude development	Qualitative		Researcher	Y			
Implementation process		Qualitative		Researcher	Y			
Staff time		Economic		Researcher			Y	
**Economic measures**								
Client service receipt inventory [35]	Information on service contacts	Economic	45 mins	Researcher			Y	
Administrative records	Cost of intervention	Economic		Researcher				Y

CQC, Care Quality Commission; QUIS, quality of interaction schedule; BL, Baseline; Pre-int, Pre-intervention; Y, Yes.

### Data management and analysis

It is planned that anonymous data and all appropriate documentation will be kept securely for a period of seven years following the completion of the trial, subject to discussion with relevant Ethics Committees.

### Quantitative data management

Administrative databases will be held at the study centre. All participants and care homes will be identified by a unique study number; this number will be used to tag all research data sent outside the study centre, for example to North Wales Organisation for Randomised Trials in Health Clinical Trials Unit (NWORTH CTU). Quantitative research data will be entered via a web interface to the MACRO research databases held at NWORTH. Primary data management will be conducted by the research team in the study centre, and the secondary cleaning and preparation of the data for analysis will be conducted by NWORTH.

### Quantitative data analysis

With the exception of the quality of interaction schedule (QUIS) observational study, which is conducted at a group level, all outcome data will be collected at an individual level for the individual participants. Outcome measures for pilot evaluation will be assessed at baseline and nine months and are listed in Table 1. All the outcome measures collected will be described and reported using appropriate descriptive statistics, tabular and graphical techniques. Particular note will be taken of baseline SDs and overall effect sizes. These data will be used to inform power calculations for Work Plan 5 (WP5). The change from baseline in the PCC group will quantified and fully described.

The CONSORT diagram information will be assessed to identify potential differences in dropout rates and other data quality issues in order to inform the design of WP5. A more detailed statistical analysis plan will be drawn up prior to analysing the data, which will be approved by the programme steering group and the Data Monitoring and Ethics committee.

The analysis of outcomes will involve a multilevel analysis of variance (ANOVA) model for the 2 × 2 × 2 factorial design with two replications among the care homes and participants, clustered within each care home. The three factors being examined are the presence or not of antipsychotic review, of social interaction and of exercise. The two replications refer to the two lists of care homes, those previously known and those not known to the investigators. Means with 95% confidence intervals will be quoted and a 5% significance level will be reported. As this is a hypotheses-generating pilot study, no adjustment will be made for multiple comparisons.

### Qualitative data management and analysis

All group discussions will be audio-recorded and transcribed verbatim. A systematic analysis procedure, based upon the principles of grounded theory, will be used to generate increased understanding of the implications of care staff attitudes for the implementation of the interventions. The aim of the analysis will be to increase the level of abstraction of themes from descriptive to interpretive. The procedure will involve at least two members of the research team independently indexing sections of the transcriptions with descriptive codes. These codes will then be organised into more abstract categories and the links between these categories identified. The categories generated in the last stage (theoretical coding) are expressed as hypotheses or propositions. Constant comparison and negative case analysis will be applied to the data from different settings to increase understanding of the processes underlying response to the interventions. Findings of the qualitative study will contribute to the optimization of the interventions, the training and the implementation approaches in the larger RCT, WP5. The qualitative study may also shed more light on specific outcomes and mediators (working mechanisms) of the interventions, as described previously. These considerations will need to be taken into account in the planned RCT.

### Regulatory and management issues

#### Ethics approval

The study was approved by Oxfordshire Research Ethics Service Committee C (REC number 11/SC/0066). Site-specific assessment (SSA) was obtained from each participating NHS Hospital Trust. The study will be conducted in accordance with the recommendations for physicians involved in research on human subjects adopted by the 18th World Medical Assembly, Helsinki 1964 and later revisions.

#### Consent

Potential participants will be identified by the managers of the participating care homes. They will seek permission from the resident and/or their personal consultee to be sent information by the research team. If permission is granted, the research team will discuss the study in further detail with the resident and/or personal consultee via telephone conversations or a meeting, depending on the preference of the individual. In addition, the relevant study information sheet will either be given to or sent to the resident and/or personal consultee. With the written consent of the participant, or the advice of the consultee, researchers will request information about diagnosis direct from GP surgeries.

The Mental Capacity Act research guidance on consent will be followed. All of the participants will be people with dementia living in the participating care homes so it is likely that a minority of potential participants will have capacity to provide informed consent. Initial conversations will take place with a potential personal consultee. If the personal consultee feels that it is appropriate for the person to take part but feels they potentially have the capacity to make their own decision, an assessment of capacity will be undertaken by a study clinician. If the individual does have capacity, a simplified written information sheet will be provided to the individual and the study will be explained to the person by a member of the research team with appropriate training and skills, where possible with the next of kin also present. If the individual wishes to take part, written consent will be taken. In addition, as a measure of good practice, signed assent will be requested from the consultee. For individuals who do not have capacity, an appropriate member of the research team will discuss the study in further detail with a consultee of the potential study participant through telephone conversations or a meeting, depending on their preference. No individuals will participate in the evaluation without signed, written consent from themselves or a signed declaration from their consultee.

All participants are free to withdraw at any time from the study without giving reasons and without prejudicing further treatment. For people who lose capacity, the consultee would be approached and the issue of ongoing participation would be discussed. If the consultee wants the person to continue to participate, the consultee will be asked to sign the declaration form. Ongoing participation would only require the completion of the nine-month outcome assessment. For the qualitative study, separate consents will be taken for staff members wishing to participate in the study FGs.

#### Confidentiality

The chief investigator will preserve the confidentiality of participants taking part in the study and is registered under the Data Protection Act (DPA, 1998). The research will follow DPA guidance. Only members of the research team will have access to the original data, which will be stored in a locked filing cabinet. Participants’ personal details will be stored separately from the original data, and will be kept in a separate file on a password-protected computer at the study centre. Each participant will be assigned an identification code, which will be used in all data storage files; these will not contain names or any other means of personal identification. All personal details will be deleted on completion of the study.

## Trial status

The trial started recruitment in May 2011 and will complete follow up assessments in 2012. Results will be reported in 2013.

## Abbreviations

ANOVA: Analysis of variance; AR: Antipsychotic review; CANE: Camberwell assessment of need for the elderly; CI: Chief investigator; CMAI: Cohen-Mansfield agitation inventory; CONSORT: Consolidated Standards of Reporting Trials; COREC: Centre of Research Ethical Campaign; CQC: Care Quality Commission; CTU: Clinical Trials Unit; DEMQOL: Measure of health-related quality of life for people with dementia; DH: Department of Health; DPA: Data Protection Act; Ex: Exercise; FAST: Functional assessment staging; FG: Focus group; FITS: Focussed intervention, training and support; GP: General Practitioner; ICC: Intra-class correlation; NDS: National Dementia Strategy; NHS: National Health Service; NICE: National Institute for Clinical Excellence; NIHR: National Institute for Health Research; NPI-NH: Neuropsychiatric Inventory - Nursing Home version; NSF: National Service Framework; NWORTH: North Wales Organisation for Randomised Trials in Health; PCC: Person-centred care; PI: Principal investigator; QoL: Quality of life; QoL-AD: Quality of life in Alzheimer’s disease; QUIS: Quality of interaction schedule; RAID: Rating Anxiety in Dementia; RCT: Randomised controlled trial; REC: Research Ethics Committee; SCIE: Social Care Institute for Excellence; SI: Social interaction; SSA: Site-specific assessment; WHELD: An optimized intervention ‘welding together’ the most effective elements of the best currently available intervention programmes and a standardised manual and training programme.

## Competing interests

The authors declare they have no competing interests.

## Authors’ contributions

CB is the chief investigator of the WHELD programme. JF and MO are site leads for the trial and, along with MK, EM-C, JM, RTW and BW-C, are principal investigators and were involved in design, grant applications, protocol development and are members of the programme management group. CB, JF and RW led the development of the protocol. RW provides statistical support to the trial and is a member of the programme management group. JS manages the trial on a day-to-day basis. All authors read and approved the final manuscript.
